# Supplementary material for: N-Glycan Modifications with Negative Charge in a Natural Polymer Mucin from Bovine Submaxillary Glands, and Their Structural Role
Source: Polymers (Basel). 2020 Dec 29;13(1):103. doi: 10.3390/polym13010103 (PMC7796149; doi:10.3390/polym13010103)
Supplement: Supplementary file 1 [file polymers-13-00103-s001.pdf]

polymers

MDPI

Supplementary Material

N-glycan Modifications with Negative Charge in a Natural Polymer Mucin from Bovine Submaxillary Glands, and Their Structural Role

Jihye Kim [1,2,†], Byoungju Lee [1,†], Junmyoung Lee [1], Minkyoo Ji [1,2], Chi Soo Park [1,2], Jaeryong Lee [1,2], Minju Kang [1,2], Jeongeun Kim [1,2], Mijung Jin [1,2] and Ha Hyung Kim [1,2,*]

[1] Biotherapeutics and Glycomics Laboratory, College of Pharmacy, Chung-Ang University, 84 Heukseok-ro, Dongjak-gu, Seoul 06974, South Korea
[2] Department of Global Innovative Drugs, Graduate School of Chung-Ang University, 84 Heukseok-ro, Dongjak-gu, Seoul 06974, South Korea
* Correspondence: hahyung@cau.ac.kr; Tel.:+82-2-820-5612
† These authors contributed equally to this work.

Received: date; Accepted: date; Published: date

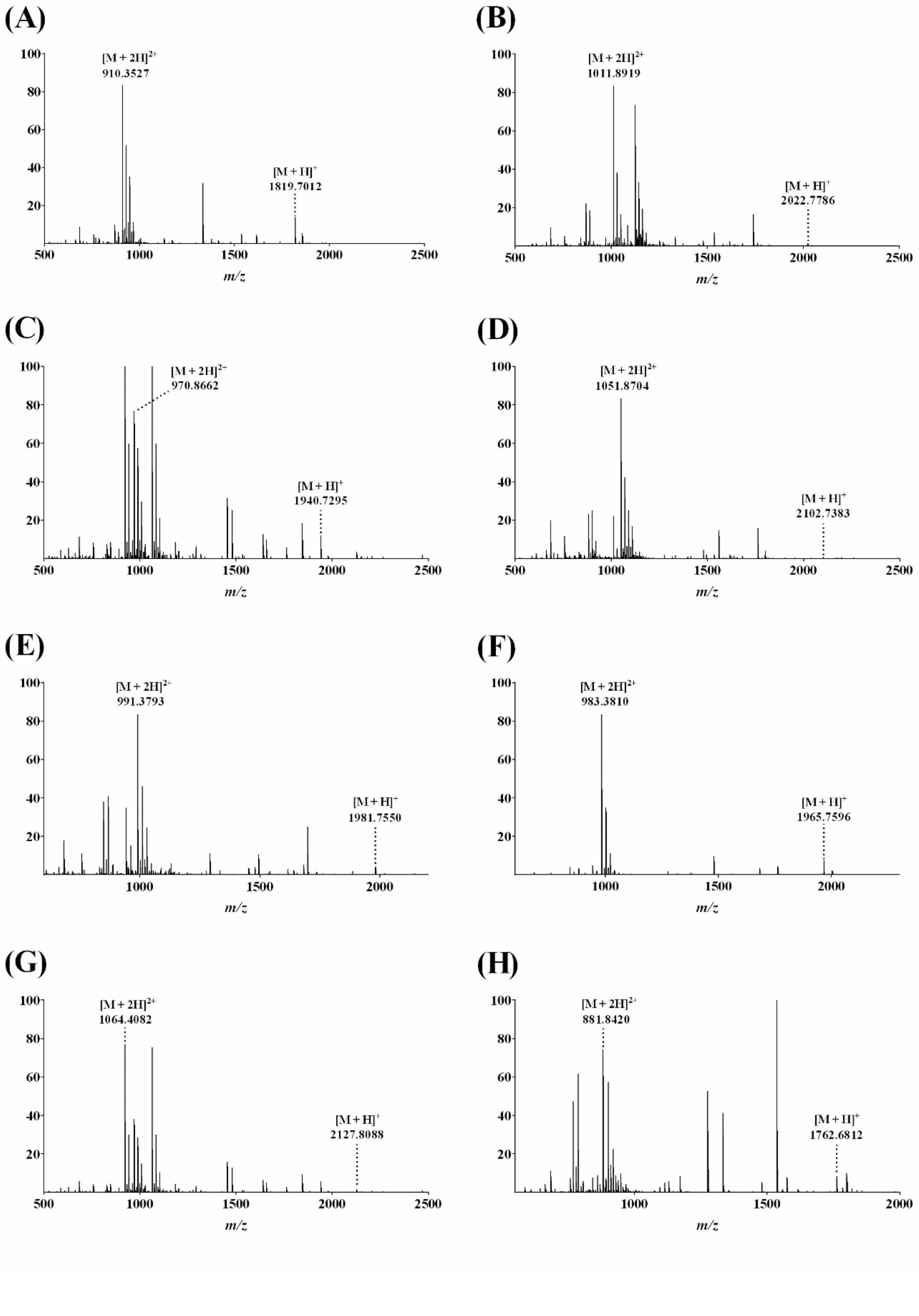
(A)
[M + 2H]2+
910.3527
[M + H]+
1819.7012
m/z
(B)
[M + 2H]2+
1011.8919
[M + H]+
2022.7786
m/z
(C)
[M + 2H]2+
970.8662
[M + H]+
1940.7295
m/z
(D)
[M + 2H]2+
1051.8704
[M + H]+
2102.7383
m/z
(E)
[M + 2H]2+
991.3793
[M + H]+
1981.7550
m/z
(F)
[M + 2H]2+
983.3810
[M + H]+
1965.7596
m/z
(G)
[M + 2H]2+
1064.4082
[M + H]+
2127.8088
m/z
(H)
[M + 2H]2+
881.8420
[M + H]+
1762.6812
m/z

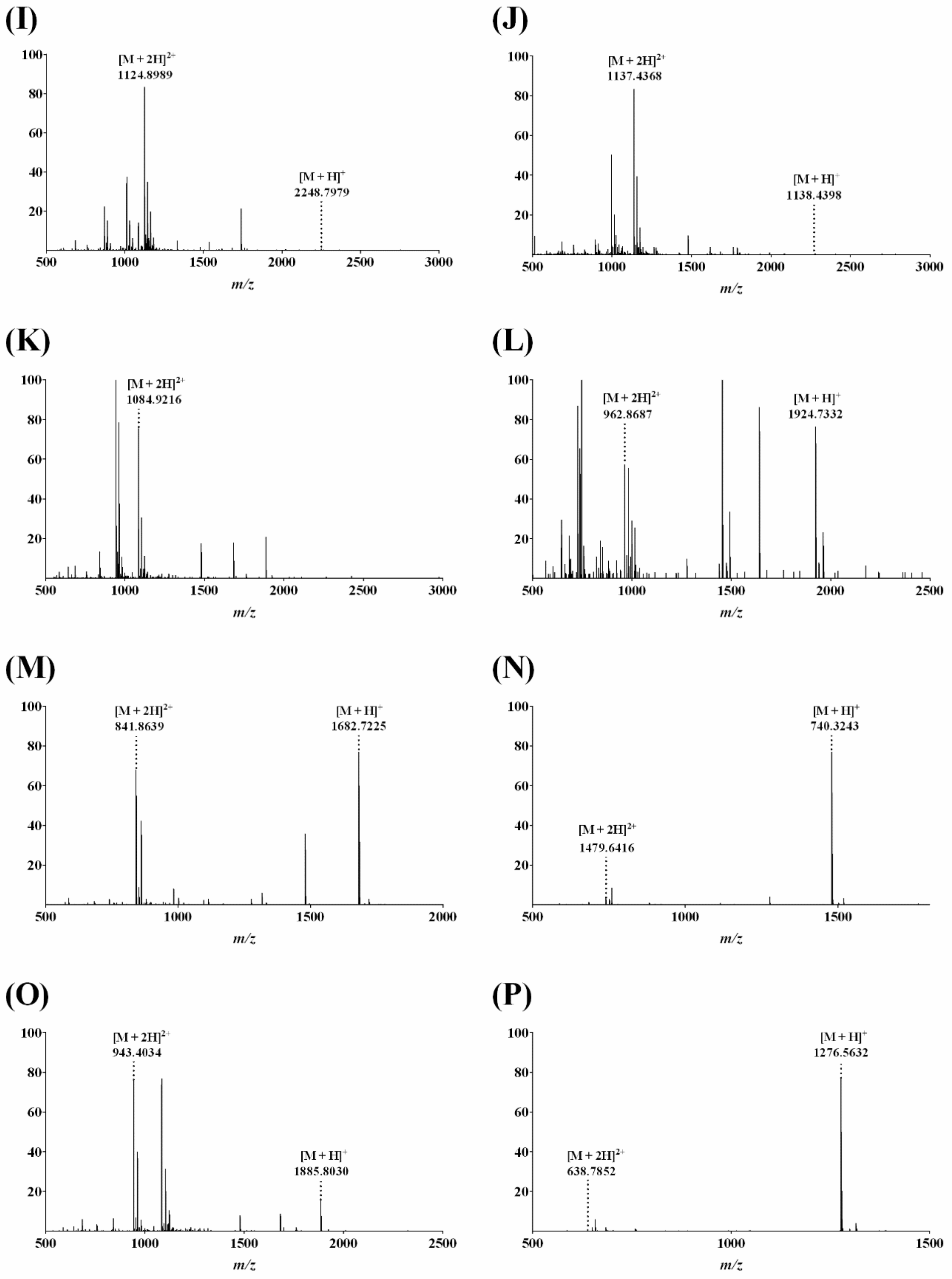
(I)
[M + 2H]2+
1124.8989
[M + H]+
2248.7979
m/z
(J)
[M + 2H]2+
1137.4368
[M + H]+
1138.4398
m/z
(K)
[M + 2H]2+
1084.9216
m/z
(L)
[M + 2H]2+
962.8687
[M + H]+
1924.7332
m/z
(M)
[M + 2H]2+
841.8639
[M + H]+
1682.7225
m/z
(N)
[M + H]+
740.3243
[M + 2H]2+
1479.6416
m/z
(O)
[M + 2H]2+
943.4034
[M + H]+
1885.8030
m/z
(P)
[M + H]+
1276.5632
[M + 2H]2+
638.7852
m/z

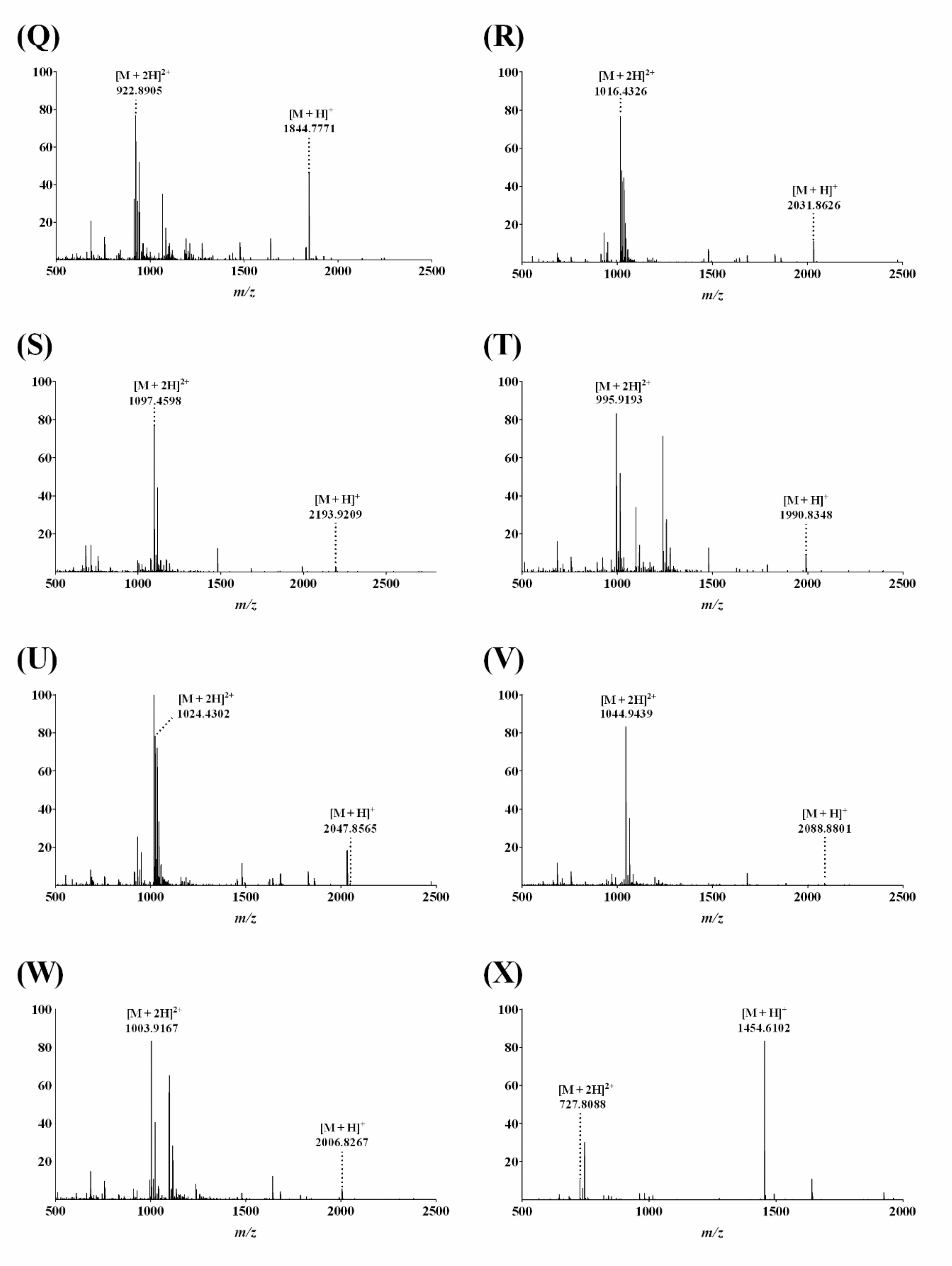
(Q)
[M + 2H]2+
922.8905
[M + H]+
1844.7771
m/z
(R)
[M + 2H]2+
1016.4326
[M + H]+
2031.8626
m/z
(S)
[M + 2H]2+
1097.4598
[M + H]+
2193.9209
m/z
(T)
[M + 2H]2+
995.9193
[M + H]+
1990.8348
m/z
(U)
[M + 2H]2+
1024.4302
[M + H]+
2047.8565
m/z
(V)
[M + 2H]2+
1044.9439
[M + H]+
2088.8801
m/z
(W)
[M + 2H]2+
1003.9167
[M + H]+
2006.8267
m/z
(X)
[M + H]+
1454.6102
[M + 2H]2+
727.8088
m/z

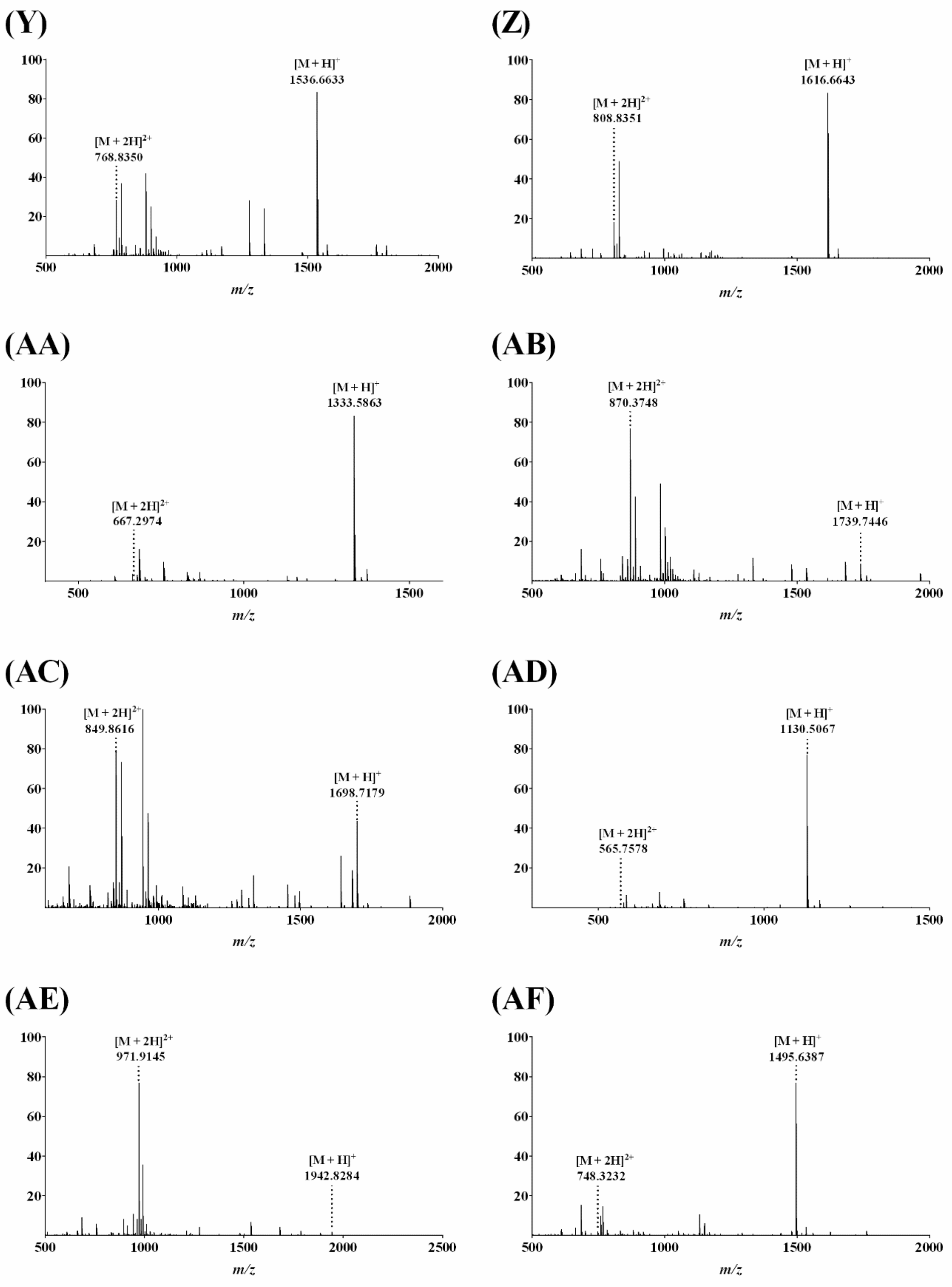

Figure S1. Full scan mass spectra of (A-E) sulfated (peaks 7, 10, 23, 9, and 18, respectively), (F-L) sulfated and core-fucosylated (peaks 13, 24, 5, 8, 27, 20, and 16, respectively), (M-W) core-fucosylated (peaks 12, 4, 19, 2, 22, 30, 32, 26, 29, 26, and 31, respectively), and (X-AF) non-core-fucosylated (peaks 15, 6, 28, 3, 14, 17, 1, 21, and 11, respectively) ProA-labeled *N*-glycans from BSM. Each peak number corresponds to a peak number in Table 1 and Figure 1. Precursor ions were detected as doubly-charged ions $[M+2H]^{2+}$ and singly-charged ions $[M+H]^{+}$.

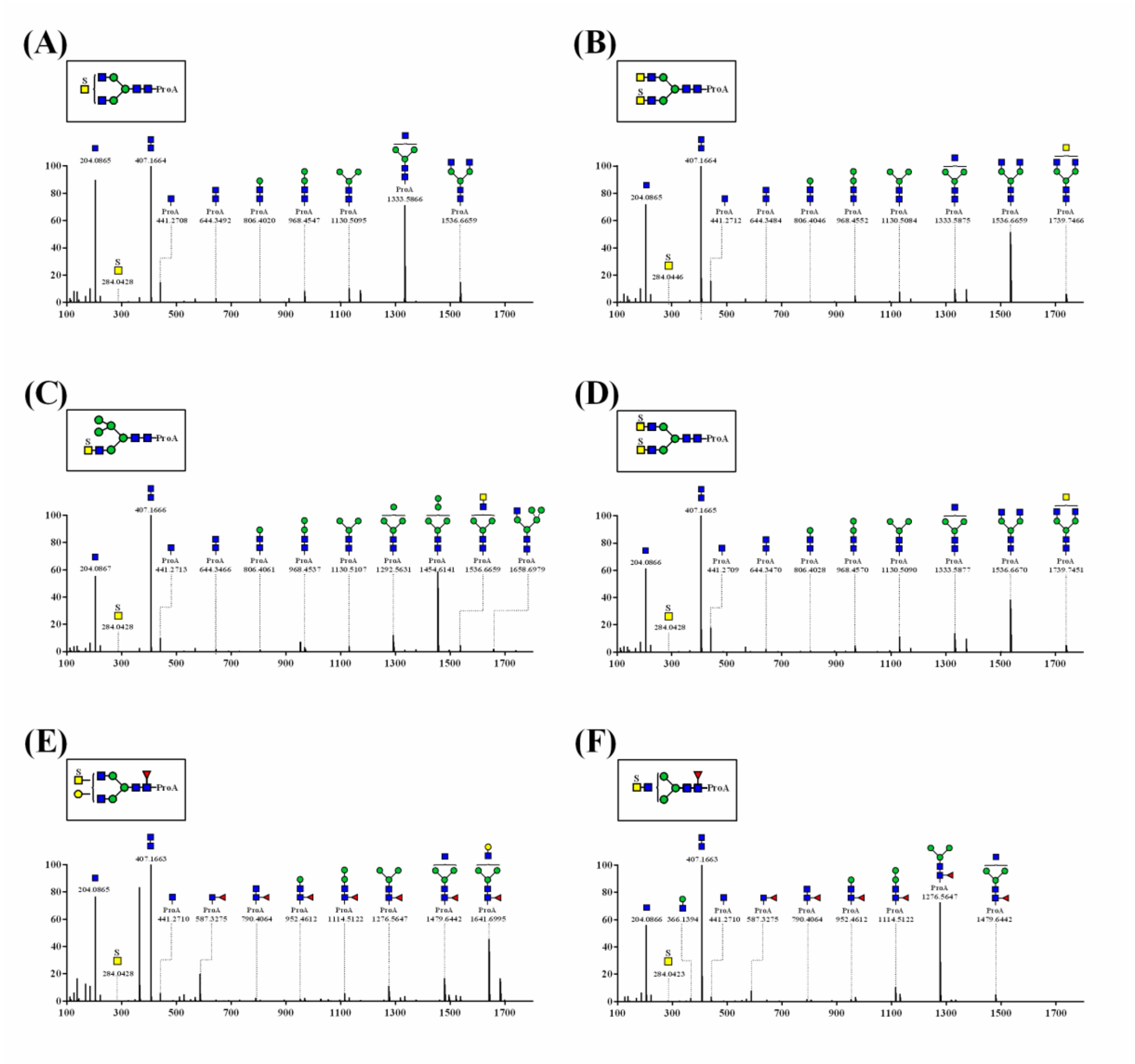
(A)
ProA
204.0865
407.1664
284.0428
441.2708
644.3492
806.4020
968.4547
1130.5095
1333.5866
1536.6659
(B)
ProA
204.0865
407.1664
284.0446
441.2712
644.3484
806.4046
968.4552
1130.5084
1333.5875
1536.6659
1739.7466
(C)
ProA
204.0867
407.1666
284.0428
441.2713
644.3466
806.4061
968.4537
1130.5107
1292.5631
1454.6141
1536.6659
1658.6979
(D)
ProA
204.0866
407.1665
284.0428
441.2709
644.3470
806.4028
968.4570
1130.5090
1333.5877
1536.6670
1739.7451
(E)
ProA
204.0865
407.1663
284.0428
441.2710
587.3275
790.4064
952.4612
1114.5122
1276.5647
1479.6442
1641.6995
(F)
ProA
204.0866
366.1394
407.1663
284.0423
441.2710
587.3275
790.4064
952.4612
1114.5122
1276.5647
1479.6442

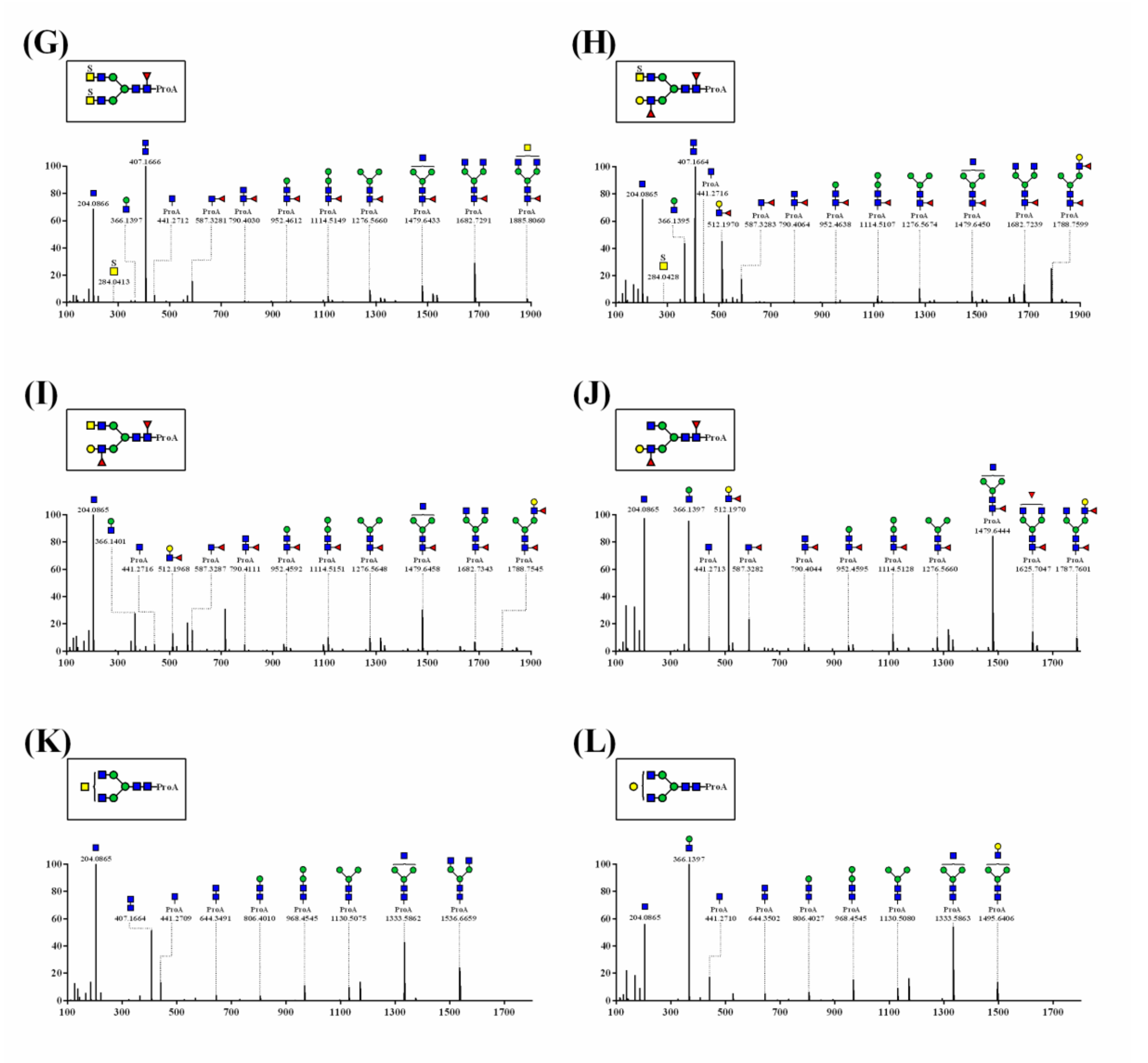

Figure S2. HCD-MS/MS spectra of (A, B, C, and D) sulfated (peaks 7, 10, 23, and 9), (E, F, G, and H) sulfated and core-fucosylated (peaks 24, 5, 8, and 27), (I and J) core-fucosylated (peaks 32 and 26), and (K and L) neutral (peaks 14 and 17) ProA-labeled *N*-glycans from BSM. Twenty *N*-glycans included 3 *N*-glycans (indicated in Figure 2) and 17 *N*-glycans (same glycan structures as in a previous report [19]) were not included. Each peak number corresponds to a peak number in Table 1 and Figure 1. Symbols: S, sulfate; ▲, Fuc; ■, GlcNAc; □, GalNAc; ○, Gal; ●, Man.
